# A comparison of the MOS-HIV and SF-12v2 for measuring health-related quality of life of men and women living with HIV/AIDS

**DOI:** 10.1186/1742-6405-8-5

**Published:** 2011-01-27

**Authors:** Allyson Ion, Wenjie Cai, Dawn Elston, Eleanor Pullenayegum, Fiona Smaill, Marek Smieja

**Affiliations:** 1 Health Research Methodology Program, Department of Clinical Epidemiology and Biostatistics, Faculty of Health Sciences, McMaster University, Hamilton, Ontario, Canada; 2 Department of Pathology and Molecular Medicine, Faculty of Health Sciences, McMaster University, Hamilton, Ontario, Canada; 3 Department of Clinical Epidemiology & Biostatistics, Faculty of Health Sciences, McMaster University, Hamilton, Ontario, Canada; 4 St. Joseph's Healthcare, Hamilton, Ontario, Canada

## Abstract

**Background:**

The purpose of this study was to examine the relationship between the Medical Outcomes Study-HIV Health Survey (MOS-HIV) and the SF-12v2 to determine if the latter is adequate to assess the health-related quality of life (HRQoL) of men and women living with HIV/AIDS. 112 men and women living with HIV/AIDS who access care at a tertiary HIV clinic in Hamilton, Ontario were included in this cross-sectional analysis. Correlation coefficients of the MOS-HIV physical and mental health summary scores (PHS and MHS) and the SF-12v2 physical and mental component summary scales (PCS and MCS) were calculated along with common sub-domains of the measures including physical functioning (PF), bodily pain (BP), general health perceptions (GH), vitality (VT), social functioning (SF) and mental health (MH) to explore the relationship between these two HRQoL measures. The sub-domains role physical (RP) and role emotional (RE) of the SF-12v2 were compared separately to the sub-domain role functioning (RF) of the MOS-HIV. Weighted kappa scores were calculated to determine agreement beyond chance between the MOS-HIV and SF-12v2 in assigning a HRQoL state (i.e. low, moderate, good, very good).

**Results:**

The MOS-HIV had mean PHS and MHS summary scores of 47.3 (SD = 11.5) and 49.2 (SD = 10.7) respectively. The mean SF-12v2 PCS and MCS scores were 47.7 (SD = 11.0) and 44.0 (SD = 10.4). The MOS-HIV and SF-12v2 physical and mental health summary scores were positively correlated (r = 0.84, p < 0.001 and r = 0.76, p < 0.001). All common sub-domains were significantly correlated at p values from < 0.001 to 0.034. Substantial agreement was observed in assigning a HRQoL state (Physical: κ = 0.788, SE = 0.095; Mental: κ = 0.707, SE = 0.095).

**Conclusions:**

This analysis validates the SF-12v2 for measuring HRQoL in adult men and women living with HIV/AIDS.

## Background

Health-related quality of life (HRQoL) measures a person's health status taking into account multiple dimensions including physical or functional, psychological and social well-being and often relies on patient self-report. Patrick and Erickson broadly define HRQoL as the "value assigned to the duration of life as modified by the impairments, functional states, perceptions, and social opportunities that are influenced by disease, injury, treatment, or policy [1]."

A paradigm shift has occurred with HIV now being considered a chronic illness due to the advancement and availability of treatment and care. Introduction of highly active anti-retroviral therapy (HAART) has resulted in a significant decrease in HIV-related morbidity and mortality across the globe; however, people living with HIV/AIDS (PHAs) continue to face a variety of health-related challenges, which can affect many aspects of their quality of life. As a result, there has been increasing interest in understanding HRQoL in the context of HIV infection across a broad spectrum of HIV research including clinical trials, observational studies and community-based research. It is important, however, to ensure that the tools used to measure HRQoL are in tune with the current state of the HIV epidemic and reflect the experience of PHAs in their local and geographical context, while minimizing the burden placed on those who participate in research studies.

Over 17 generic and HIV-specific HRQoL measures are used in HIV research today and there is no consensus on which measures are best, especially considering that many of these measures were developed in the pre-HAART era [2]. In a comparative review by Clayson et al., the SF-36 was identified as the generic measure with the greatest evidence supporting its use in HIV/AIDS research [2]. The Medical Outcomes Study HIV Health Survey (MOS-HIV) was identified as one of the preferred HIV-specific measures since it is brief and practical to administer, the input of PHAs was used in its development, there is well-established evidence for its reliability, validity and responsiveness and it has been successfully used in clinical trials [2]. Shahriar et al. countered Clayson's review stating that there was insufficient evidence to recommend the use of the MOS-HIV over the SF-36 and that more head-to-head comparisons were needed [3].

The MOS-HIV is a 35-item questionnaire that includes eleven dimensions of HRQoL including general health perceptions (GHP), bodily pain (BP), physical functioning (PF), role functioning (RF), social functioning (SF), mental health (MH), energy/vitality (EV), cognitive functioning (CF), health distress (HD), overall quality of life (QL) and health transition (HT) allowing for the generation of physical (PHS) and mental (MHS) health summary scores. Development of the MOS-HIV began in 1987 and items selected from the SF-20 were the foundation for its construction [3-5]. The MOS-HIV was developed to provide a brief, comprehensive measure of functional status and well-being of PHAs enrolled in large-scale clinical trials and has been shown to be internally consistent and responsive to a number of outcomes including infections, adverse events, increased symptoms and AIDS-related events [2,4,5]. The MOS-HIV has also been used in studies with a variety of patient groups including treatment-naïve, asymptomatic PHAs to those with more advanced HIV and opportunistic infections. MOS-HIV items are rescaled to a number between 0 and 100, with a higher score reflecting better health and HRQoL [4-6].

The 12-item short-form (SF-12v2) health survey, now in its second version, was developed out of a strategy to construct a shorter version of the SF-36 Health Survey reflecting the same sub-domains including general health perceptions (GHP), bodily pain (BP), physical functioning (PF), role physical (RP), role emotional (RE), social functioning (SF), mental health (MH) and energy/vitality (EV) [4,7]. The SF-12v2 reproduces more than 90% of the variance of the physical and mental component summary scales of the SF-36 in the general US population, takes significantly less time to complete than the SF-36, reducing burden on research participants; and demonstrated high two-week test-retest reliability correlations for both the physical (r = 0.89) and mental (r = 0.76) health summary scores [6,8]. Han et al. demonstrated the SF-12v2 to be a reasonable and effective replacement for the SF-39, a similar measure to the MOS-HIV, in studies of people living with advanced HIV disease by comparing five domains of the SF-12 (namely physical functioning, general health perceptions, bodily pain, mental health and energy/fatigue) to the SF-39 [9]. This analysis demonstrated that the burden of data requirements for both participants and investigators as well as redundancy of questions asked could be reduced by using the SF-12v2 [8].

The purpose of this study was to give further rationale for using the SF-12v2 in HIV research by examining the relationship between the MOS-HIV and the SF-12v2 to determine if, when compared to the HIV-specific MOS-HIV, the SF-12v2 is an adequate measure to assess the health-related quality of adult men and women living with HIV/AIDS.

## Methods

The study population consisted of 112 adult men and women living with HIV/AIDS who accessed care at the McMaster University Medical Centre Special Immunology Services outpatient clinic in Hamilton, Ontario and were enrolled in the Canadian HIV Vascular Study, a multi-centre, prospective cohort study examining the relationship between HIV infection, anti-retroviral therapy and cardiovascular disease. The Canadian HIV Vascular Study was approved by the Hamilton Health Sciences/McMaster University Faculty of Health Sciences Research Ethics Board; all participants gave their informed consent prior to their inclusion in this study and analysis of their data. MOS-HIV and SF-12v2 questionnaires completed on the same day during the Canadian HIV Vascular Study baseline interview were used. The MOS-HIV served as the reference standard as it is the primary HIV-specific HRQoL measure used in clinical and observational HIV research; there is no evidence that the SF-39, a similar HRQoL scale, has ever been used in HIV research. The continuous PHS and MHS of the MOS-HIV and the PCS and MCS of the SF-12v2 were assessed for normality. Correlations between baseline physical and mental health summary scores of both measures were calculated using SPSS v17; Pearson correlation coefficients were calculated because of the lack of skew in the distributions of the summary scores. Pearson correlation coefficients were used to investigate the relationship between common sub-domains of the MOS-HIV and SF-12v2 including physical functioning (PF), bodily pain (BP), general health perceptions (GH), energy/vitality (VT), social functioning (SF) and mental health (MH). The sub-domains role physical (RP) and role emotional (RE) of the SF-12v2 were compared separately to the domain role functioning (RF) of the MOS-HIV as these two domains capture the overall "role functioning" measured in the MOS-HIV. Pearson correlation coefficients and the Multitrait-Multimethod Matrix method as outlined by Campbell and Fiske [10] were used to assess convergent and discriminant validity. Convergent validity indicates the degree to which sub-domains of the measures are related whereas discriminant validity indicates to what extent the sub-domains are not related theoretically; both convergent and discriminant validity were assessed statistically [11]. A cut-off of r ≥ 0.70 was chosen to determine the degree of convergent validity [12,13]; a cut-off of r < 0.85 was chosen to assess discriminant validity [11].

We also investigated agreement between the two measures in assigning individuals to a HRQoL state, for example, low, moderate, good and very good HRQoL. Quartile values of the PHS and MHS from the MOS-HIV generated out of descriptive statistics of the cohort were used to establish levels of low (PHS: 0-39.09; MHS: 0-41.03), moderate (PHS: 39.10-48.47; MHS: 41.04-49.89), good (PHS: 48.48-57.34; MHS: 49.90-58.50) and very good (PHS: 57.35-100; MHS: 58.51-100) HRQoL. SF-12v2 PCS and MCS quartile values were calculated for low (PCS: 0-41.02; MCS: 0-36.79), moderate (PCS: 41.03-51.35; MCS: 36.80-44.44), good (PCS: 51.36-56.27; MCS: 44.45-52.69) and very good (PCS: 56.28-100; MCS: 52.70-100) HRQoL and were compared to the MOS-HIV quartiles for each individual generating a 4 x 4 table. Weighted kappa (κ) scores as per Fleiss and Cohen [14] were calculated using software by Cyr and Francis [15] in order to determine the chance-corrected agreement between the MOS-HIV and SF-12v2 in assigning individuals to levels of HRQoL. Weighted kappa values were interpreted as follows: less than 0 -- poor agreement; 0 to 0.2 -- slight agreement; 0.2 to 0.4 -- fair agreement; 0.4-0.6 -- moderate agreement; 0.6-0.8 -- substantial agreement; 0.8-1.0 -- almost perfect agreement [16].

A secondary analysis was conducted using the baseline clinical and HRQoL data from 96 of the men and women in the cohort from whom we had complete baseline data in order to determine the clinical validity of the SF-12v2 compared to the MOS-HIV. Pearson correlation coefficients were calculated in univariable analysis for all clinical variables of interest with each HRQoL summary score from both measures. Four linear regression models were created in SPSSv17 utilizing the physical health and mental health summary scores of both the SF-12v2 and MOS-HIV as outcome measures. The overall fit of each model was assessed and standardized beta coefficients for each clinical variable of interest were reviewed for statistical significance and contribution to the model. The following clinical variables were included in each regression model: age, gender, years living with HIV, smoking (current and former), current marijuana use, drug use (including cocaine and heroin), current receipt of a NNRTI-based or PI-based HAART regimen, nadir CD4 cell count and average number of hours slept each night. These variables were chosen because they have shown to affect physical or mental HRQoL in the literature [17-28].

## Results

Table 1 presents baseline characteristics of the 112 men and women living with HIV/AIDS who were included in the analysis. The cohort was predominantly male with a mean age of 49.1 years (SD = 8.2) and Caucasian ethnicity. The HIV transmission risk factor cited most frequently was sex with other men (61.6%) followed by heterosexual/bisexual sex (29.5%) and injection drug use (6.3%). The cohort had a mean CD4 T-lymphocyte count of 507 cells/ml of blood at their baseline study visit (SD = 280.3) and had lived with HIV, on average, for 12.0 years (SD = 7.6). Table 2 presents the descriptive statistics for the physical and mental health summary scores as well as all domains of the MOS-HIV and SF-12v2. The mean MOS-HIV physical health summary score was 47.3 (SD = 11.5) ranging from 22.4 to 63.2 whereas the mean MOS-HIV mental health summary score was 49.2 (SD = 10.7) ranging from 20.5 to 66.7. The mean physical and mental component summary scales of the SF-12v2 were similar at 47.7 (SD = 11.0) ranging from 16.2 to 63.4 and 44.0 (SD = 10.4) ranging from 16.7 to 62.4, respectively.

**Table 1:** Baseline characteristics of participants

N = 112	Mean (SD); Min-Max
Age (years)	49.1 (8.2); 31-75

Number of years living with HIV	12.0 (7.6); 1-52

Baseline CD4 (at study visit)	507.4 (280.3); 50-1170

	**N (%)**

Gender	
*Male*	97 (86.6)
*Female *	14 (12.5)
*Transgendered*	1 (0.9)

Currently receiving HAART	87 (77.7)

Ethnicity	
*Caucasian*	100 (89.3)
*Black*	7 (6.3)
*Other*	3 (2.7)
*First Nation*	2 (1.8)

HIV Transmission Risk Factor	
*MSM*	69 (61.6)
*Heterosexual/Bisexual*	33 (29.5)
*IDU*	7 (6.3)
*Hemophilia*	5 (4.5)
*Blood Products*	3 (2.7)
	

**Table 2:** Mean, Standard Deviation, Median and Min-Max Values for Components/Domains of MOS-HIV and SF-12v2 (n = 112)

MOS-HIV				SF-12v2			
**Component or Domain**	**Mean (SD)**	**Median**	**Min-Max**	**Component or Domain**	**Mean (SD)**	**Median**	**Min-Max**

PHS	47.3 (11.5)	48.5	22.4-63.2	PCS	47.7 (11.0)	51.3	16.2-63.4

MHS	49.2 (10.7)	49.9	20.5-66.7	MCS	44.0 (10.4)	44.4	16.7-62.4

GHP	48.9 (10.6)	48.3	28.9-67.6	GHP	45.4 (11.4)	44.7	18.9-62.0

BP	50.8 (9.1)	50.6	27.6-62.2	BP	46.0 (11.9)	47.3	16.7-57.4


PF	47.8 (11.2)	51.2	20.2-58.1	PF	47.3 (11.4)	52.2	22.1-56.5

RF	48.0 (10.8)	56.6	32.0-56.6	RP	46.4 (10.6)	48.0	20.3-57.2
				RE	44.6 (12.2)	44.9	11.3-56.1

SF	46.5 (11.6)	47.8	10.2-57.2	SF	43.7 (11.2)	46.5	16.2-56.6

MH	50.7 (11.7)	53.6	17.5-66.3	MH	43.3 (10.0)	46.3	15.8-64.5

EV	47.4 (11.6)	48.9	24.3-68.6	EV	49.3 (9.9)	47.7	27.6-67.9

CF	46.3 (10.9)	48.3	14.1-58.1				

HD	51.8 (10.5)	53.7	20.4-62.0				

QL	49.2 (9.8)	53.0	27.7-65.6				

Abbreviations: PHS - physical health summary score; MHS - mental health summary score; PCS - physical component summary scale; MCS - mental component summary scale; GHP - general health perceptions; BP - bodily pain; PF - physical functioning; RF - role functioning; RP - role physical; RE - role emotional; SF - social functioning; MH - mental health; EV - energy/vitality; CF - cognitive functioning; HD - health distress; QL - quality of life

Table 3 presents correlation coefficients computed comparing the physical and mental health summary scores of the MOS-HIV and SF-12v2 as well as scores of all sub-domains in each measure. The MOS-HIV and SF-12v2 were positively correlated with regard to both the physical and mental health summary scores respectively (r = 0.84, p < 0.001 and r = 0.76, p < 0.001). A comparison of the MOS-HIV and SF-12v2 common domains including PF, BP, GH, VT, SF and MH yielded positive correlations for all categories (PF: r = 0.90; BP: r = 0.82; GH: r = 0.80; VT: r = 0.72; SF: r = 0.68; MH: r = 0.58; all significant at p < 0.001). The domains role physical and role emotional of the SF-12v2 were compared separately to the domain role functioning of the MOS-HIV yielding slightly lower, yet positive correlations (RP: r = 0.69; RE: r = 0.49; p < 0.001). Tables 4 and 5 present the inter-domain correlations of the SF-12v2 and MOS-HIV, respectively. Five of the inter-scale correlations of the SF-12v2 were low (r range = 0.24-0.39), however, the remaining correlations were moderately to highly associated (r range = 0.40-0.86, all statistically significant at p values from < 0.001 to 0.012). Inter-scale correlations of the MOS-HIV were similar with moderate to high inter-scale correlations ranging from 0.40 to 0.70, all statistically significant at p < 0.001. The two exceptions were the associations between the PF and MH (r = 0.36) and between GH and CF (r = 0.39). Overall, by comparing the Pearson correlations between the measures as well as the inter-domain correlations within the SF-12v2 and MOS-HIV to the cut-off values of r≥0.70 and r < 0.85 chosen, it was demonstrated that both instruments have good convergent and discriminant validity, respectfully.

**Table 3:** Correlation between SF-12v2 and MOS-HIV (n = 112)

	MOS-HIV											
	
	PF	PN	GH	VT	SF	MH	RF	CF	QL	HD	PHS	MHS
SF12v2												

PF	0.9	0.68	0.63	0.61	0.41	0.31	0.62	0.41	0.47	0.56	0.84	0.52

BP	0.62	0.82	0.5	0.52	0.43	0.37	0.46	0.36	0.36	0.55	0.7	0.49

GH	0.56	0.52	0.8	0.64	0.43	0.41	0.47	0.2	0.58	0.45	0.66	0.56

VT	0.54	0.48	0.6	0.72	0.39	0.36	0.43	0.36	0.54	0.37	0.62	0.55

SF	0.4	0.46	0.44	0.5	0.68	0.64	0.37	0.4	0.53	0.5	0.52	0.65

MH	0.45	0.49	0.57	0.76	0.53	0.58	0.43	0.52	0.57	0.51	0.59	0.71

RP	0.78	0.67	0.7	0.7	0.53	0.39	0.69	0.38	0.55	0.5	0.85	0.58

RE	0.44	0.49	0.47	0.66	0.55	0.68	0.49	0.63	0.46	0.64	0.56	0.75

PCS	0.85	0.74	0.69	0.57	0.4	0.24*	0.6	0.25	0.46	0.49	0.84	0.44

MCS	0.27*	0.36	0.45	0.69	0.58	0.71	0.36	0.56	0.54	0.52	0.44	0.76

SF12: PF = physical functioning; BP = bodily pain; GH = general health perceptions; VT = vitality; SF = social functioning; MH = mental health; RP = role physical; RE = role emotional; PCS = physical component summary scale; MCS = mental component summary scale

MOS-HIV: PF = physical functioning; PN = pain; GH = general health perceptions; VT = energy/fatigue; SF = social functioning; MH = mental health; RF = role functioning; CF = cognitive function; QL = quality of life; HD = health distress; HT = health transition; PHS = physical health summary; MHS = mental health summary

Note: All correlations were statistically significant at p < 0.001 except for *, which were significant at p < 0.05.

**Table 4:** Inter-domain correlations within SF-12v2 (n = 112)

	PF	BP	GH	VT	SF	MH	RP	RE	PCS	MCS
PF		0.66*	0.50*	0.54*	0.37*	0.45*	0.77*	0.39*	0.89*	0.21+

BP			0.47*	0.42*	0.41*	0.44*	0.62*	0.39*	0.80*	0.26+

GH				0.59*	0.40*	0.55*	0.54*	0.33*	0.66*	0.41*

VT					0.24*	0.86*	0.59*	0.45*	0.49*	0.65*

SF						0.40*	0.43*	0.55*	0.35*	0.61*

MH							0.51*	0.59*	0.36*	0.84*

RP								0.54*	0.82*	0.37*

RE									0.25+	0.83*

PCS										0.08

MCS										

SF12: PF = physical functioning; BP = bodily pain; GH = general health perceptions; VT = vitality; SF = social functioning; MH = mental health; RP = role physical; RE = role emotional; PCS = physical component summary scale; MCS = mental component summary scale

*: Statistically significant (p < 0.001)

+: Statistically significant (p < 0.05)

**Table 5:** Inter-domain correlations within MOS-HIV (n = 112)

	PF	PN	GH	VT	SF	MH	RF	CF	QL	HD	PHS	MHS
PF		0.67	0.69	0.67	0.50	0.36	0.64	0.43	0.56	0.58	0.90	0.58

PN			0.61	0.66	0.46	0.47	0.54	0.49	0.47	0.63	0.81	0.61

GH				0.68	0.51	0.47	0.64	0.39	0.62	0.60	0.82	0.68

VT					0.62	0.62	0.57	0.50	0.62	0.61	0.80	0.79

SF						0.70	0.44	0.48	0.58	0.56	0.66	0.76

MH							0.40	0.60	0.50	0.68	0.48	0.89

RF								0.47	0.46	0.55	0.81	0.58

CF									0.41	0.70	0.52	0.77

QL										0.45	0.64	0.71

HD											0.65	0.85

PHS												0.72

MHS												

MOS-HIV: PF = physical functioning; PN = pain; GH = general health perceptions; VT = energy/fatigue; SF = social functioning; MH = mental health; RF = role functioning; CF = cognitive function; QL = quality of life; HD = health distress; HT = health transition; PHS = physical health summary; MHS = mental health summary

Note: All p values are < 0.001

The MOS-HIV and SF-12v2 demonstrated substantial agreement for assigning individuals to specific states of HRQoL based on their MOS-HIV physical and mental health summary scores with weighted κ scores of 0.788 (SE = 0.095) and 0.707 (SE = 0.095) for agreement of physical and mental health, respectively.

Lastly, the univariable and multivariable analyses investigating clinical correlates of HRQoL between the SF-12v2 and MOS-HIV demonstrated moderate agreement (Table 6). There was similar directionality and magnitude of association between the two measures for both the physical and mental health summary scores. In univariable analysis, a history of drug use was associated with a lower physical health summary score for both the MOS-HIV [r = - 0.216 (95% CI - 0.399, - 0.017)] and SF-12v2, however the correlation was not significant for the SF-12v2 [r = - 0.157 (95% CI - 0.346, 0.044)]. The MOS-HIV and SF-12v2 mental health summary scores demonstrated similar trends with regard to male gender [MOS-HIV: r = 0.222 (95% CI 0.023, 0.404); SF-12v2: r = 0.164 (95% CI - 0.037, 0.352)] and hours slept each night [MOS-HIV: r = 0.194 (95% CI - 0.006, 0.379); SF-12v2: r = 0.207 (95% CI 0.007, 0.391)]. In multivariable analysis, the trend for the MHS was maintained for male gender (MOS-HIV: β = 0.260, p = 0.013; SF-12v2: β = 0.199, p = 0.052) and hours slept each night (MOS-HIV: β = 0.283, p = 0.011; SF-12v2: β = 0.270, p = 0.014). The one discrepancy between the two measures was with regard to smoking history and the mental health summary score. In univariable analysis, the mental health summary score of both measures was not significantly correlated with being a current smoker [MOS-HIV: r = - 0.011 (95% CI - 0.211, 0.189); SF-12v2: r = 0.044 (95% CI - 0.157, 0.242)] or former smoker [MOS-HIV: r = - 0.014 (95% CI -0.213, 0.187); SF-12v2: r = 0.048 (95% CI -0.153, 0.246)]. In multivariable analysis, current smoker and former smoker were significant predictors of the MOS-HIV MHS (β = 4.226, p = 0.044; β = -4.25, p = 0.043, respectively), but not of the SF-12v2 MCS (β = 1.867, p = 0.363; β = -1.865, p = 0.364, respectively), even though directionality of the associations were similar. It should be noted that only the regression model involving the SF-12v2 MCS as the dependent variable was statistically significant (F = 1.955, p = 0.044). The other regression models were not significant: SF-12v2 PCS -- F = 0.924, p = 0.522; MOS-HIV MHS: F = 1.735, p = 0.80; MOS-HIV PHS: F = 1.352, p = 212.

**Table 6:** Correlation coefficients (95% CIs) and multivariable regression (standardized beta coefficients) exploring correlates of HRQoL

Clinical variable of interest	MOS-HIV	SF-12v2	MOS-HIV	SF-12v2
	Physical health summary score (PHS)	Physical component summary scale(PCS)	Mental health summary score (MHS)	Mental component summary scale(MCS)
	r (95% CI)	r (95% CI)	r (95% CI)	r (95% CI)
	β (p value)	β (p value)	β (p value)	β (p value)
Age	r = -0.011	r = -0.067	r = 0.128	r = 0.228
	(-0.211, 0.189)	(-0.767, 0.543)	(-0.074, 0.320)	(0.029, 0.409)
	β = -0.063 (p = 0.579)	β = -0.116 (p = 0.321)	β = 0.153 (p = 0.169)	β = 0.215 (p = 0.052)

Male Gender	r = 0.155	r = 0.115	r = 0.222	r = 0.164
	(-0.046, 0.344)	(-0.087, 0.308)	(0.023, 0.404)	(-0.037, 0.352)
	β = 0.174 (p = 0.099)	β = 0.102 (p = 0.345)	β = 0.260 (p = 0.013)	β = 0.199 (p = 0.052)

Years living w HIV	r = -0.117	r = -0.166	r = -0.059	r = 0.197
	(-0.310, 0.085)	(-0.354, 0.035)	(-0.143, 0.256)	(-0.003, 0.382)
	β = -0.163 (p = 0.161)	β = -0.213 (p = 0.076)	β = -0.016 (p = 0.886)	β = 0.130 (p = 0.246)

Current smoker	r = -0.113	r = -0.069	r = -0.011	r = 0.044
	(-0.306, 0.089)	(-0.265, 0.133)	(-0.211, 0.189)	(-0.157, 0.242)
	β = -0.973 (p = 0.646)	β = -1.279 (p = 0.556)	β = 4.226 (p = 0.044)	β = 1.867 (p = 0.363)

Former smoker	r = -0.105	r = -0.064	r = -0.014	r = 0.048
	(-0.299, 0.097)	(-0.261, 0.138)	(-0.213, 0.187)	(-0.153, 0.246)
	β = 0.869 (p = 0.681)	β = 1.240 (p = 0.568)	β = -4.254 (p = 0.043)	β = -1.865 (p = 0.364)

Currently uses marijuana	r = -0.099	r = -0.065	r = -0.032	r = -0.045
	(-0.293, 0.103)	(-0.262, 0.137)	(-0.231, 0.169)	(-0.243, 0.156)
	β = -0.186 (p = 0.098)	β = -0.097 (p = 0.397)	β = -0.128 (p = 0.244)	β = -0.143 (p = 0.187)

Has used drugs (including cocaine and heroin)	r = -0.216	r = -0.157	r = -0.119	r = -0.103
	(-0.399, -0.017)	(-0.346, 0.044)	(-0.312, 0.083)	(-0.297, 0.099)
	β = -0.199 (p = 0.081)	β = -0.129 (p = 0.266)	β = -0.179 (p = 0.109)	β = -0.094 (p = 0.392)

Currently receiving PI-based regimen	r = 0.016	r = -0.027	r = 0.084	r = 0.137
	(-0.185, 0.215)	(-0.226, 0.174)	(-0.118, 0.279)	(-0.065, -0.128)
	β = 0.067 (p = 0.582)	β = 0.025 (p = 0.843)	β = 0.117 (p = 0.326)	β = 0.157 (p = 0.185)

Currently receiving NNRTI-based regimen	r = 0.177	r = 0.141	r = 0.125	r = 0.181
	(-0.024, 0.364)	(-0.061, 0.332)	(-0.077, 0.317)	(-0.020, 0.368)
	β = 0.087 (p = 0.479)	β = 0.116 (p = 0.357)	β = 0.006 (p = 0.961)	β = 0.050 (p = 0.672)

Hours slept each night	r = 0.078	r = -0.008	r = 0.194	r = 0.207
	(-0.124, 0.274)	(-0.208, 0.192)	(-0.006, 0.379)	(0.007, 0.391)
	β = 0.141 (p = 0.209)	β = -0.018 (p = 0.877)	β = 0.283 (p = 0.011)	β = 0.270 (p = 0.014)
Nadir CD4 cell count	r = -0.057	r = -0.067	r = -0.093	r = -0.045
	(-0.254, 0.154)	(-0.263, 0.135)	(-0.288, 0.109)	(-0.767, -0.543)
	β = -0.018 (p = 0.883)	β = -0.080 (p = 0.515)	β = -0.018 (p = 0.880)	β = 0.112 (p = 0.336)

## Discussion

This preliminary analysis suggests that the SF-12v2 is an appropriate measure of health-related quality of life of men and women living with HIV/AIDS compared to the MOS-HIV demonstrating high correlation and good convergent and discriminant validity when compared to the physical and mental health summary scores of the MOS-HIV and common sub-domains. Furthermore, the SF-12v2 had substantial agreement with the MOS-HIV in assigning individuals to a specific HRQoL status and determining clinically relevant correlates of HRQoL.

It is important to point out that this analysis does not account for the HRQoL domains of cognitive functioning, health distress and health transition, which are captured in the MOS-HIV but are not represented in the SF-12v2. These domains are used to derive the mental health summary score of the MOS-HIV, which may help to explain the weaker correlation between the measures in the MHS as well as the differences in determining clinically relevant correlates of HRQoL. If the SF-12v2 is used as a HRQoL measure in any HIV research study, it would have to be with the caveat that these three HRQoL domains were not important outcomes or were not relevant to the population under study.

It should be noted that the mean physical and mental health summary scores were lower than the mean score of 50 for the reference population. This supports the literature that despite the advancement of HAART and decline in HIV-related morbidity and mortality, people living with HIV continue to experience health-related challenges and generally have lower physical and mental HRQoL scores when compared to the general population. A cross-sectional questionnaire-based study conducted by Miners et al. found that men and women living with HIV in the United Kingdom scored lower on all five domains on the EQ-5D quality of life measure including mobility, self-care, usual activities, pain/discomfort and anxiety/depression irrespective of similarities in age and gender [22]. Univariable and subsequent multivariable regression analysis demonstrated that people living with HIV had significantly lower utility and visual analogue scale scores on the EQ-5D compared with the general population; HIV infection independently decreased the utility and visual analogue scale scores of the EQ-5D by 20% [22]. In addition, the mean mental health summary scores were relatively higher for people completing the MOS-HIV compared to the SF-12v2. This may reflect the additional domains captured in the MOS-HIV (i.e. health distress, health transition, etc.) that are combined to determine the mental health summary score or may have arisen due to chance.

The SF-12v2 is currently being used in HIV research in Canada to better understand the HRQoL of individuals living with HIV/AIDS including assessing changes over time, but had not been formally compared to the MOS-HIV. The Canadian HIV Vascular Study investigators chose the SF-12v2 over the MOS-HIV in order to reduce questionnaire burden on participants, and the SF-12v2 is also being used in the Ontario HIV Treatment Network Cohort Study to understand yearly changes in HRQoL. The SF-12v2 is a contemporary HRQoL measurement tool with accessible language and efficiency in its administration. Ease in reading and comprehending the SF-12v2 would also result in fewer errors by the participant.

Although this is not necessarily synonymous with the level of understanding of the intended meaning of the items, anecdotally, the authors have experienced minimal issues in interpreting the SF-12v2, but have often had questions from participants completing the MOS-HIV, including redefinition of colloquial language such as "pep," "blue" and "down in the dumps." The MOS-HIV typically takes much longer to complete than the SF-12v2. Locally, participants involved in research at the McMaster University Medical Centre usually need 5 to 10 minutes to complete the MOS-HIV, whereas individuals can usually complete the SF-12v2 in less than 2 minutes and express ease in completing the SF-12v2 more so than the MOS-HIV. Miscomprehension of terms used in HRQoL measures by participants can result in inaccurate measurement of this important construct. It is important to use a HRQoL measure that is culturally relevant, accessible, quick to administer and reflects the current experiences of PHAs. It should be acknowledged that it was not possible to ask participants directly regarding the 'burden' or time required to complete both the MOS-HIV and SF-12v2. This would have offered an interesting perspective to this analysis and the subsequent decision of which measure to use in HIV research studies. Another consideration when measuring HRQoL is to what extent the items and dimensions captured in the scale resonate with participants and accurately depict the current reality of PHAs. It was not possible to elicit feedback from PHAs via focus groups or in-depth interviews prior to inclusion of the MOS-HIV and SF-12v2 in this analysis; this would have offered another interesting perspective to this comparison.

This analysis may not be generalizable to all PHAs. The cohort was comprised predominantly of men with an average age of 48.6 years (ranging from 31 to 75 years) whose major HIV transmission risk factor was intercourse with other men; the study sample is reflective of the early HIV epidemic and may not be comparable to today's population of people living with HIV/AIDS. Eighty-nine per cent were of Caucasian ethnicity and only 12.6% of the cohort were women, therefore, caution should be taken when attempting to apply these results to people from different ethnocultural communities and gender identities. These findings must also be considered with caution due to the relatively small sample size.

## Conclusions

This preliminary analysis suggests that the SF-12v2 is an efficient and practical HRQoL questionnaire taking, on average, less than two minutes to complete. This HRQoL measure may enable timely collection of quality of life data in broader areas of research than in the past while reducing the redundancy and questionnaire burden placed on participants. Confirmatory studies in larger and more representative populations are needed.

## Competing interests

The authors declare that they have no competing interests.

## Authors' contributions

AI and MS conceived the design of the study, performed and interpreted the statistical analysis and helped to draft the manuscript. FS participated in the design of the study and helped to draft the manuscript. DE and WC participated in the coordination of the study, assisted with the statistical analysis and helped to draft the manuscript. EP assisted with development and interpretation of the statistical analysis and helped to draft the manuscript. All authors read and approved the final manuscript.
